# Fever of unknown origin (FUO) revised

**DOI:** 10.1007/s00508-016-1083-9

**Published:** 2016-09-26

**Authors:** Manuel Unger, Georgios Karanikas, Andreas Kerschbaumer, Stefan Winkler, Daniel Aletaha

**Affiliations:** 1 Division of Rheumatology, Department of Medicine 3, Medical University of Vienna, Waehringer Guertel 18–20, 1090 Vienna, Austria; 2 Division of Nuclear Medicine, Department of Biomedical Imaging and Image-Guided Therapy, Medical University of Vienna, Vienna, Austria; 3 Division of Infectious Diseases and Tropical Medicine, Department of Medicine 1, Medical University of Vienna, Vienna, Austria

**Keywords:** Fever of unknown origin, Diagnostics, ^18^FDG-PET/CT, Naproxen test, Sarcoidosis

## Abstract

Fever of unknown origin (FUO) was originally characterised in 1961 by Petersdorf and Beeson as a disease condition of temperature exceeding 38.3 °C on at least three occasions over a period of at least three weeks, with no diagnosis made despite one week of inpatient investigation. However, since underlying diseases are often reported for classical FUO, these presentations may not be considered to be of “unknown origin”. Rather, the aetiology of prolonged fever may resolve, or not resolve. The definition of fever with unresolved cause (true FUO) is difficult, as it is a moving target, given the constant advancement of imaging and biomarker analysis. Therefore, the prevalence of fever with unresolved cause (FUO) is unknown.

In this review, we report such a case of prolonged fever, which initially has presented as classical FUO, and discuss current literature. Furthermore, we will give an outlook, how a prospective study on FUO will allow to solve outstanding issues like the utility of different diagnostic investigations, and the types and prevalence of various underlying diseases.

## Case report

We present a 46-year-old man with a classic case of fever of unknown origin (FUO), who visited the outpatient department of the division of Infectious Diseases and Tropical Medicine in December 2015. His main complaint was a sustaining fever up to 39.2 °C, which was not subsiding since three weeks despite two different antibiotics prescribed by the general practitioner. He reported distinctive b symptoms with night sweats and incipient weight loss, as well as emerging dyspnoea.

Routine blood tests showed no significant abnormalities, whereas a chest radiograph indicated bihiliary lymphadenopathy, leading to the differential diagnoses of tuberculosis, lymphoma, or possibly sarcoidosis. Therefore, a computed tomography was performed, which on the one hand confirmed the finding of the enlarged lymph nodes, and on the other hand showed no sign of tuberculous cavity. Interferon-gamma-release assay (IGRA) testing for tuberculosis was also negative. Furthermore, angiotensin-converting enzyme determined in the serum was highly elevated, making the diagnosis of sarcoidosis much more likely than lymphoma. In parallel, fever disappeared immediately under treatment with naproxen, which was another indication for a nonmalignant cause of fever.

Already on the third day after admission, mediastinoscopy and a consecutive lymph node biopsy were performed. Histologic examination confirmed the diagnosis of sarcoidosis.

## Review

### Definitions and history of original FUO

In 1961, Petersdorf and Beeson defined fever of unknown origin (FUO) as a state of febrile illness for more than three weeks, with a body temperature greater than 38.3 °C on several occasions and uncertain diagnosis after one week of study in hospital. [1] The considerations behind it were to eliminate self-limiting infectious diseases and “habitual hyperthermia” as well as allowing execution of the—at the time—lengthy diagnostic workup.

Later in 1991, Durack and Street initiated two major changes to the original definition [2]. First, they recommended differentiating between four classes of FUO: classical FUO as originally defined by Petersdorf and Beeson, nosocomial FUO, neutropenic FUO, and HIV-associated FUO. This separation of HIV-related disease from other causes of fever is plausible as its treatment is clearly more time critical. Furthermore, Durack and Street proposed a minimum diagnostic evaluation of three outpatient visits or three days of in-hospital investigation, before classifying a case as FUO. This was meant to allow for the time needed for incubating blood cultures and tuberculosis skin tests to become positive. With the advent of modern diagnostic methods, such as polymerase chain reaction (PCR) and computed tomography (CT), Knockaert and Vanderschueren updated the definition of FUO once again [3]. They proposed to change the quantitative criterion of three days’ investigation after which no diagnosis has been made to a qualitative one, by claiming for an “appropriate intelligent standard inpatient or outpatient workup”.

In the meanwhile, even more sophisticated diagnostic tools, such as ^18^fluoro-deoxy-glucose–positron emitted tomography (^18^FDG-PET), have entered clinical routine over the course of the last decade, and many biochemical and serological assays have become routinely available. While in clinical routine they have become an attractive tool for diagnostic evaluation of patients with prolonged fever, it is unclear to what extent they may facilitate a diagnosis in patients who have already been evaluated by a number of other tests.

### Aetiology and epidemiology of original FUO

Even with modern diagnostic tools and advanced therapeutic possibilities, despite—and maybe even because of—its low prevalence, FUO remains to be one of the unresolved challenges in medicine. Original FUO may be a symptom of approximately 200 described causes [4–6]. Existing literature suggests a classification of causes of classical FUO into four main aetiological categories: infections, neoplasms, non-infectious inflammatory diseases (NIID; e. g. connective-tissue diseases, vasculitides), and miscellaneous conditions (Table 1) [4]. Some authors regard undiagnosed or idiopathic FUO as a fifth category, accounting for around 10–50 % of cases [1, 7–10]. In fact, this last category is the truly fever of unknown origin.

As can be seen in Table 1, infections were the leading cause for classical FUO in most published studies, accounting for approximately one third of all cases: abscesses, endocarditis, tuberculosis, and complicated urinary tract infections dominate the group of infection-related FUO, regardless of the age of the patient. In comparison to this, in the group of NIID, mostly rare diseases are represented, such as Still’s disease and systemic lupus erythematosus as the most frequent causes seen in younger patients, and temporal arteritis and polymyalgia rheumatica in the elderly. In this older age group, NIID, even though rare themselves, are even more frequent causes of FUO than infections [11]. Among patients with neoplasms, FUO can be related to the tumour itself (approximately one third of patients, e. g. to lymphoma), or more frequently by complicating infections (two thirds) [12]. Aetiology also clearly depends on geography, as in developing countries the percentage of infections is much higher than in developed countries, while for neoplasms and NIID it is the opposite.

**Table 1:** Causes of classic fever of unknown origin ^1^NIID, Non-infectious inflammatory disease

Author (pub. year)	Infection (%)	Neoplasm (%)	NIID^1^ (%)	Misc. (%)	Undiagnosed (%)
Petersdorf (1961) [1]	36	19	19	19	7
Larson (1982) [7]	30	31	16	11	12
Knockaert (1992) [6]	22.5	7	23	21.5	26
De Kleijn (1997) [9]	26	12	25	8	30
Bleeker-Rovers (2007) [10]	16	7	22	4	51

However, it is surprising that despite the improvement of diagnostic technologies, especially imaging modalities, some studies still indicate an increased percentage of undiagnosed FUO [13].

In terms of prognosis, overall, 12–35 % of patients were reported to die from diseases underlying classical FUO [7, 14]. Mortality depends on the nature of the underlying disease, and prognosis is worse if diagnostic delay occurs [4]. Especially patients with undiagnosed FUO (unresolved cause) have overall a good prognosis, usually with resolution of their fever in four or more weeks; the 5‑year mortality rate lies around 3.2 %, and between 51 and 100 % of these patients are reported to have spontaneous recovery.

### Diagnostic approach

Several approaches with a staged diagnostic protocol have been published [10, 13, 15–17]. They suggest individualizing the algorithms, as it is obviously impossible to perform all tests on all patients. There is also common agreement that a detailed patient history and physical examination is crucial in patients presenting with an unclear febrile illness. It has been subject of debate, if documenting specific fever patterns provide information on diagnosis, although characteristic patterns, such as the Pel-Ebstein type in case of Hodgkin’s disease, have reported diagnostic value [18]. Otherwise, with the possible exception of the tertian and quartan patterns of malaria, these fever patterns are neither sensitive nor specific enough to be considered diagnostic of any disease.

Following this basic diagnostic approach, there is a controversy about the next step. This includes questions about which laboratory tests are necessary to rule out common conditions; which imaging investigations are needed; at which point in time is the use of more invasive diagnostic tests justified; are there potential rare but dangerous differential diagnoses that require a more time critical evaluation; etc. Most authors claim a rather broad panel of standardised tests, including a large number of blood tests, urinalysis, stool tests, skin test, cultures from different material, chest X‑ray and ultrasonography of upper abdomen. If no diagnosis is achieved, it is proposed to perform another series of tests: hepatitis B serology, anergy test, IgD in serum, liver biopsy, culture for *Mycobacteria,* other bacteria and fungi; crista biopsy; echocardiography; CT of abdomen and chest; X‑ray of the colon and temporal artery biopsy in patients over 55 years [13, 15].

In 2007 Bleeker-Rovers et al. added the term “potentially diagnostic clue” (PDC) to the diagnostic algorithm, meaning to follow a diagnostic suspicion that presents itself after an initial basic workup. This requires a directed and personalised approach to additional investigations in order to verify or rule out the diagnostic suspicion [10].

With the advent of ^18^FDG-PET in routine clinical care, the diagnostic workup of FUO has been further expanded, allowing the imaging of acute and chronic inflammatory processes by uptaking FDG in all activated leukocytes, i. e. granulocytes, monocytes and lymphocytes [19]. Especially in combination with CT, the resulting ^18^FDG-PET/CT provides the necessary spatial resolution and may substantially contribute in finding the cause of FUO [20, 21]. It is obvious that this makes the ^18^FDG-PET/CT attractive for inclusion in the routine diagnostic workup of patients with FUO, although given the radiation exposure and the cost, the position in a diagnostic algorithm needs yet to be determined.

### Therapeutic approach

Empiric therapy is not recommended in patients with prolonged fever, because it may camouflage and therefore delay diagnosis, and subsequently also hamper correct treatment decisions. There are only a few exceptions when treatment needs to be initiated solely based on a primary diagnostic suspicion: antibiotics for suspected culture-negative endocarditis; tuberculostatics in suspected active tuberculosis; and glucocorticoids for suspected temporal arteritis with the risk of vision loss [22]. Particularly, the empirical use of glucocorticoids is only required in highly exceptional cases [23].

Antipyretic therapy as symptomatic approach is a special form of empiric therapy, as it does not necessarily require understanding the cause of fever. Despite its camouflage of the presenting symptom, it may be necessary in a considerable proportion of patients who are not tolerating the fever or fever peaks. For diagnostic purposes, although lacking sufficient sensitivity and specificity (see below) it may still be good to understand the degree, type and pattern of fever. In patients with known cancer, antipyretic therapy has been reported to discriminate between fever from the underlying malignant disease (in case of response) and fever from concomitant infections (in case of nonresponse) [24, 25]. If this also applies to fever in individuals without an established underlying diagnosis is controversial. A typical example are reports on the so-called “naproxen test”, first described in 1984 by Chang et al. [26] The authors elaborate on the usefulness of naproxen in the differential diagnosis of FUO. Patients with FUO for more than seven days were treated with naproxen: while patients with infectious fever showed no response, patients with underlying neoplastic condition showed a prompt lysis of fever within 24 h after the initiation of naproxen. While Chang insists on the value of the test [27], Vanderschueren et al. postulated its lack of sensitivity and specificity in distinguishing neoplasm-related fevers from those caused by infections. However, their conclusion was somewhat limited as selection of naproxen in this study was at the discretion of the physician, as were timing, dosage and duration of administration. By testing not only naproxen, but also other nonsteroidal anti-inflammatory drugs (NSAIDs), Vanderschueren and colleagues pointed out that since the antipyretic mechanism of NSAIDs is independent of the aetiology of the fever, none of them is helpful in the differential diagnosis of prolonged fever. In addition, earlier results on the efficacy of naproxen might have been biased, because patients with obvious infections were excluded from analysis in these studies [28]. Since then, the naproxen test is the subject of controversial discussions. However, since for the control of fever NSAIDs are sufficient in most patients, ignoring a potential diagnostic value would be a waste. Particularly in the context of a broader diagnostic picture, i. e. by considering multiple diagnostic clues, such as biomarker and imaging investigations, a series of valuable tests may create clinical relevant likelihood ratios for diagnosis.

## Design of a prospective study: FEBRIX

To prospectively address the open questions (as partly discussed above), we designed a specific study protocol. In this FEBRIX study, patients with prolonged fever (at least one week; orally measured temperature ≥37.8 °C) will be recruited over a 2-year period. These sensitive entry criteria will allow the evaluation of the utility of different diagnostic investigations, and to determine the types and prevalence of various underlying diseases. Consequently, the prevalence of fever with unresolved cause (FUO) will also be investigated. The evaluation process will be based on a diagnostic algorithm, which includes diagnostic therapies (naproxen test) and modern imaging techniques (^18^FDG-PET/CT). At the end of the study, a large battery of clinical, serological, and biomarker analyses from material obtained at baseline and during follow-up will be linked to the outcome of the prolonged fever (cause resolved or not resolved) with the purpose of predicting outcomes of future patients with prolonged fever at an early point of their disease.

**Fig. 1:**
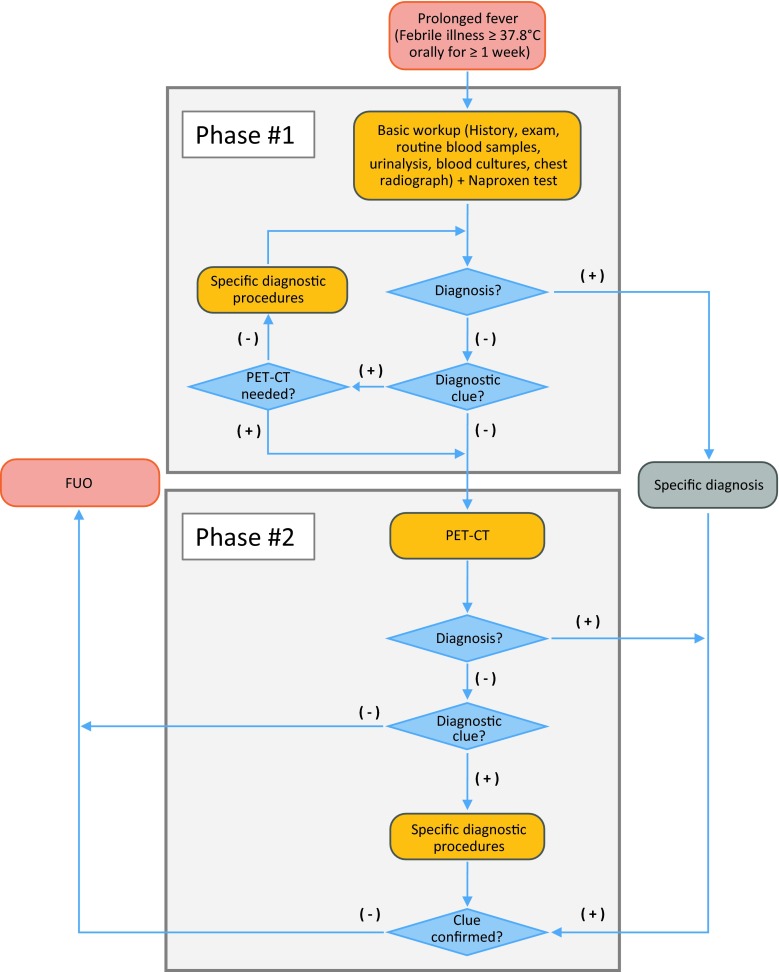
Proposed algorithm for a diagnostic approach to prolonged fever: Patients with a febrile illness ≥37.8 °C (orally) for ≥1 week will undergo a basic clinical workup during Phase #1 of the study (*top box*). If no diagnosis can be established during this initial phase, or no clue regarding a potential differential diagnosis can be made, patients enter Phase #2. There an ^18^FDG-PET/CT will be performed to elicit further diagnostic clues (*bottom box*). *FUO* fever of unknown origin

The diagnostic algorithm (Fig. 1) consists of two phases, lasting for a maximum of 2 weeks. The examinations that will be performed in Phase #1 of the study will—as in clinical routine—be guided by clinical suspicion and decisions of the treating specialists at the Division of Infectious Diseases and the Division of Rheumatology of the Medical University of Vienna, located at the Vienna General Hospital. A minimum basic diagnostic workup, however, will contain a detailed history, physical examination, routine peripheral blood evaluations, including aerobic and anaerobic blood cultures, urinalysis and a standard chest X‑ray. In case of referral of a patient from outside the Vienna General hospital—again aligned with routine clinical care—tests, for which there is no documentation, or which are older than 5 days, will be repeated as possible. Antipyretics will be discontinued at presentation to appropriately assess the extent, type and course of fever. Phase #1 will also include the evaluation of a diagnostic treatment intervention using naproxen (see above).

Two physicians will be required to document the implications of the initial test results: to establish a diagnosis (i. e. resolve the cause of prolonged fever) consensus is needed among these two; this diagnosis will be documented with the supporting evidence. If no diagnosis is evident at this point, the presence of a diagnostic clue will be evaluated: a diagnostic clue would be a clinical suspicion of underlying cause of prolonged fever, which in itself does not prove a diagnosis, but results in additional, more specific diagnostic examinations or procedures. Here, the two physicians are not required to achieve consensus, but rather by involving two physicians the sensitivity for a primary diagnostic clue will be increased. Documentation at this point will therefore include (1) the diagnostic suspicion or suspicions; and (2) the finding(s), which triggered this suspicion. Physicians will also be instructed to document if the naproxen test provided a diagnostic clue.

If no diagnostic clue is present or a physician requests an ^18^FDG-PET/CT, then patients directly enter Phase #2 of the study, in which they will receive an ^18^FDG-PET/CT evaluation. Based on the ^18^FDG-PET/CT, a diagnosis may be established, or new diagnostic clues may arise, leading to additional diagnostic procedures. If no diagnosis can be established, then FUO (prolonged fever with unresolved cause) is present.

After conclusion of the study, the prevalence of the different outcomes will be analysed: FUO vs. prolonged fever with specific underlying diagnosis (malignancy, infection, or noninfectious inflammatory disease). A post hoc analysis of clinical markers and biomarkers at baseline will then be performed to identify potential predictors of outcome at study entry, which may help modify/optimise clinical approach to patients with prolonged fever as well as FUO in the future.
